# Collapsing Glomerulopathy in a Patient With Malaria and Multiple Myeloma: A Case Report

**DOI:** 10.1155/carm/9149610

**Published:** 2026-08-10

**Authors:** Moritz Harenberg, Kerstin Amann, Daniel Zickler

**Affiliations:** ^1^ Department of Nephrology and Medical Intensive Care, St. Joseph Krankenhaus, Berlin, Germany; ^2^ Department of Nephropathology, Department of Pathology, University of Erlangen-Nürnberg, Erlangen, Germany, fau.eu

## Abstract

Collapsing glomerulopathy is a severe form of focal segmental glomerulosclerosis (FSGS) that can occur as a primary or secondary condition, often in the context of infections such as HIV. Multiple myeloma can cause kidney injury through mechanisms such as cast nephropathy, while malaria‐associated kidney injury is most often due to acute tubular necrosis. We present the case of a 47‐year‐old male of African descent with well‐controlled HIV infection who became critically ill with severe AKI requiring intensive care and renal replacement therapy. A blood smear confirmed *Plasmodium falciparum* infection, and the clinical course was further complicated by a previously undiagnosed multiple myeloma. Given the multiple potential etiologies, a kidney biopsy was performed, which ruled out myeloma‐associated kidney injury and revealed collapsing glomerulopathy. The temporal association with the malaria infection, together with the well‐controlled HIV status, pointed to malaria‐associated collapsing glomerulopathy as the most likely diagnosis. This case highlights the diagnostic challenges of AKI in critically ill patients with overlapping comorbidities and underscores the value of kidney biopsy in guiding the differential diagnosis.

## 1. Background

FSGS is one of the leading causes of end‐stage kidney disease. It can be classified as either primary (idiopathic) or secondary, with the secondary forms often associated with other underlying diseases or conditions [1]. Collapsing glomerulopathy, a distinct histological variant of FSGS, is characterized by collapse of the capillaries of the glomerulus. It is commonly associated with HIV as HIV‐associated nephropathy (HIVAN), but it can also occur secondary to other infections, including malaria and recently also in the form of SARS‐CoV‐2‐associated nephropathy (COVAN) [2, 3]. Genetic factors, notably the APOL1 gene risk variants, have been linked to an increased incidence of collapsing glomerulopathy in individuals of African descent [4].

Malaria, caused by *Plasmodium* species, remains a leading cause of morbidity and mortality worldwide, particularly in sub‐Saharan Africa. Severe malaria can lead to complications such as hemolysis, jaundice, and AKI. The typical histopathologic finding of malaria‐associated AKI in the setting of *P. falciparum* infection is acute tubular necrosis (ATN), which often results from hemodynamic instability [5, 6]. However, there are also other pathogenic mechanisms described in malaria‐associated AKI, such as microvascular sequestration of parasitized erythrocytes causing renal ischemia, intravascular hemolysis with release of nephrotoxic cell‐free hemoglobin and a dysregulated systemic inflammatory response [7]. Apart from ATN, glomerulonephritis has also been observed in malaria‐associated kidney injury. Acute, reversible glomerulonephritis—typically membranoproliferative or mesangioproliferative—can occur with *P. falciparum* infection, while chronic, progressive immune complex–mediated glomerulonephritis, historically termed quartan malarial nephropathy (QMN), is classically associated with *P. malariae* infection and may progress to end‐stage kidney disease [8, 9].

Multiple myeloma (MM), a plasma cell malignancy, is another well‐known cause of kidney injury. The most common form of kidney disease in MM is cast nephropathy, in which monoclonal light chains obstruct renal tubules [10]. AL amyloidosis represents a less common cause of myeloma‐associated AKI compared to cast nephropathy, characterized by predominantly glomerular accumulation of amyloid‐forming light chain proteins that classically presents with heavy albuminuria [11].

In critically ill patients, diagnosing the cause of AKI can be particularly challenging, as multiple mechanisms may be involved [12, 13]. This case report discusses the complexities of diagnosing collapsing glomerulopathy in a patient with malaria and MM.

## 2. Case Presentation

A 47‐year‐old male, originally from Cameroon and residing in Germany, presented to an outside hospital with a 3‐day history of high‐grade fever, coughing, and malaise. He had a known history of HIV, for which he was on antiretroviral therapy (ritonavir, dolutegravir, and darunavir), with an undetectable viral load. The patient also had well‐controlled arterial hypertension (treated with candesartan) and chronic kidney disease KDIGO Stage 2 (baseline creatinine of 1.5 mg/dL and eGFR [CKD‐EPI] 64 mL/min). The etiology of the pre‐existing CKD was unknown; only two prior creatinine values were available. His last visit to Cameroon was 6 months prior to admission.

Upon admission to an outside hospital, the patient was found to be lethargic but with stable vital signs. Initial blood tests revealed anemia (hemoglobin 8.6 g/dL), thrombocytopenia (42/nL), hyponatremia (124 mmol/L), and elevated inflammatory markers (CRP 192 mg/L). The patient was diagnosed with Stage 3 AKI (creatinine 4.57 mg/dL), and urinalysis showed significant proteinuria (3+), along with hematuria (3+). A chest x‐ray showed bilateral lung opacities, leading to a presumptive diagnosis of community‐acquired pneumonia complicated by sepsis. Empiric treatment with piperacillin/tazobactam was started, after blood cultures were collected.

However, the following day, the patient’s anemia and kidney failure worsened, and hemolysis was found, prompting further investigation. Given the constellation of fever, hemolysis, thrombocytopenia, and AKI, hemolytic uremic syndrome (HUS) was considered, leading to his transfer to our hospital as a major nephrologic referral center for further evaluation and treatment.

**FIGURE 1:**
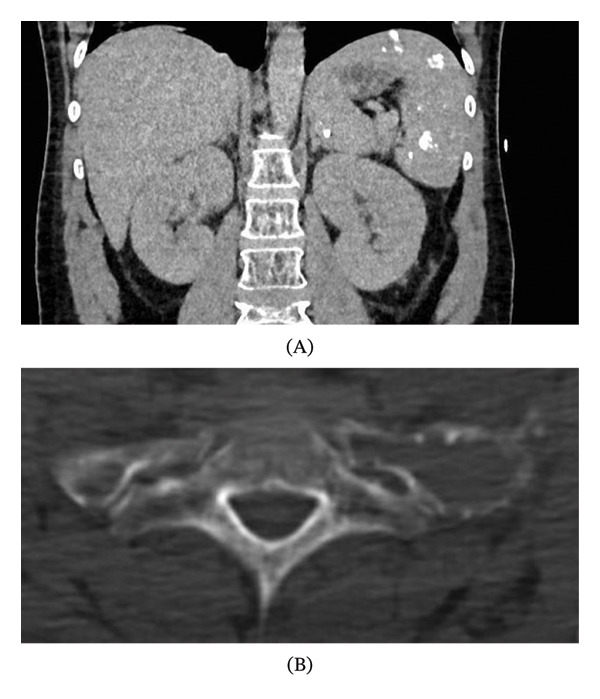
(A) Contrast‐enhanced CT of the abdomen, portal venous phase. The kidneys demonstrate edematous swelling and decreased corticomedullary differentiation. Incidentally noted are splenic calcifications. (B): Noncontrast CT of the cervical spine. A large lytic lesion of the left posterior first rib with a soft tissue component is noted.

Upon transfer to our hospital, the patient exhibited pancytopenia, Coombs‐positive hemolysis, and markedly elevated inflammatory markers. Renal function continued to decline rapidly with a marked rise in serum creatinine to 10.3 mg/dL. CT showed edematous kidneys with decreased corticomedullary differentiation (Figure 1A). Significant proteinuria was detected with a urine protein‐to‐creatinine ratio (UPCR) of 6.1 g/g, almost exclusively albuminuria. Complement levels (C3 and C4) were severely reduced.

Given the worsening clinical condition, plasma exchange was prepared but not initiated, as fragmentocytes were not detected on peripheral blood smear. In the context of fever, hemolysis, severe AKI, and recent travel to sub‐Saharan Africa, a blood smear was performed, which confirmed *Plasmodium falciparum* infection with a parasitemia of 1%. This led to a diagnosis of complicated malaria, and intravenous artesunate was started for 3 days, followed by oral artemether/lumefantrine for 5 days. The blood smear was negative for *Plasmodium* on Day 4 of treatment. This treatment resulted in resolution of hemolysis and fever, although renal function continued to worsen, and anuria developed.

On Day 4, kidney replacement therapy was initiated due to progressive renal failure. Although complicated malaria was considered a likely cause of AKI, other etiologies remained a concern, especially due to the severe proteinuria. A comprehensive serologic workup revealed abnormal immunofixation with an IgG kappa precipitation band. The kappa free light chain level was significantly elevated at 10,900 mg/L, with a free light chain ratio of 289. CT imaging of the neck revealed multiple osteolytic lesions in the cervical spine, raising suspicion for MM (Figure 1B).

A bone marrow aspiration was performed, revealing 26% myeloma cells in the flow cytometry analysis. This confirmed the diagnosis of MM. Treatment for MM was initiated with bortezomib and dexamethasone 5 days after the initial presentation.

**FIGURE 2:**
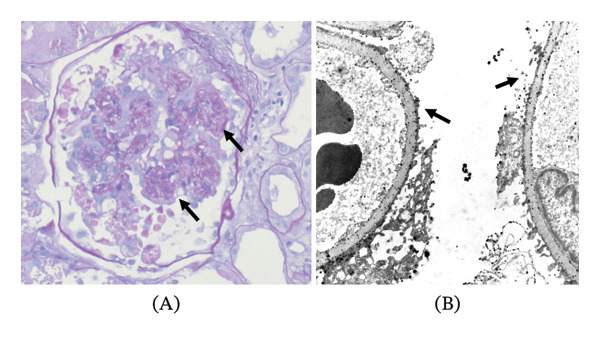
Kidney biopsy. Light microscopy ((A); PAS stain, x40) showing a glomerulus with partially collapsed capillaries (arrows). Electron microscopy ((B), x5000) showing severe foot process effacement of the affected podocytes (arrows).

Given the complex presentation, a kidney biopsy was performed 13 days after initial presentation to clarify the cause of the progressive renal injury. The biopsy revealed collapsing glomerulopathy in 3 out of 11 glomeruli, along with severe tubular injury and tubular dilatation (Figure 2). Interstitial fibrosis and tubular atrophy were rated as 5%. No evidence of cast nephropathy or light chain deposition was found, ruling out a myeloma‐related kidney disease. There were no signs of amyloidosis on light microscopy, and Congo red staining was negative.

The initial pathological interpretation suggested HIVAN, given the patient’s known HIV status. However, upon further review and considering the well‐controlled HIV infection, the findings were more consistent with malaria‐associated kidney disease. This highlights the importance of considering the full clinical context when interpreting renal biopsy results.

The patient continued kidney replacement therapy for 6 weeks, with gradual improvement in renal function. By the time of discharge, his serum creatinine had improved to 2.5 mg/dL, and he no longer required dialysis. At follow‐up at 12 weeks, the creatinine was stable. The UPCR, however, was unchanged at around 7 g/g. The MM was managed with chemotherapy, including daratumumab, bortezomib, lenalidomide, and dexamethasone. After induction therapy, the patient received high‐dose chemotherapy with subsequent autologous stem cell transplant, followed by daratumumab maintenance therapy. At follow‐up after 1 year, the creatinine was still stable and the UPCR reduced to 1.2 g/g.

## 3. Discussion

This case underscores the complexity of diagnosing the cause of AKI in critically ill patients with multiple comorbidities. Initially, the patient’s presentation suggested a sepsis‐induced AKI, a common cause of kidney failure in critically ill patients [14]. However, despite appropriate sepsis management, renal function did not improve, which prompted further investigation.

The presence of hemolysis raised the possibility of thrombotic microangiopathy (TMA) [15]. However, the positive Coombs test and lack of fragmentocytes excluded this diagnosis. Given the patient’s travel history, malaria was considered, and a blood smear confirmed *Plasmodium falciparum* infection. While malaria‐related AKI is typically associated with ATN, this patient presented with glomerular injury, characterized by significant proteinuria. In this scenario of unclear disease mechanism and multiple differential diagnoses, a kidney biopsy remains an important diagnostic tool [16].

The biopsy in this case revealed collapsing glomerulopathy which represents a distinct and aggressive variant of FSGS, characterized by higher serum creatinine, more severe proteinuria, and poorer renal outcomes compared to classical FSGS [17].

As collapsing glomerulopathy is the hallmark of HIVAN, the combination of known HIV infection and typical histological finding initially prompted this diagnostic consideration. However, several factors argued against HIVAN as the etiology of the kidney injury in this case. The patient had a longstanding diagnosis of HIV and was under constant antiretroviral therapy with undetectable viral load, a setting in which the development of HIVAN is highly unlikely [18]. Furthermore, the baseline kidney function was only mildly reduced, whereas the acute kidney injury coincided with the *Plasmodium falciparum* infection.

A review of the literature showed that malaria‐associated collapsing glomerulopathy has been increasingly recognized in recent years. After isolated case reports [19, 20], a comprehensive retrospective study in France of kidney biopsies performed in the context of malaria found collapsing FSGS to be the most common histological finding in patients with malaria‐associated kidney injury [21]. Notably, all but one of the 23 patients in that cohort were of African ancestry, and all seven patients who underwent APOL1 genotyping carried high‐risk variants. This striking predilection for individuals of African descent likely reflects the role of APOL1 as a key genetic susceptibility factor. The APOL1 G1 and G2 risk variants, which rose to high allele frequency in sub‐Saharan African populations due to the selective advantage they confer against trypanosomal infection, increase the risk of kidney diseases such as FSGS and HIVAN [22]. However, only a minority of individuals with two APOL1 risk alleles develop kidney disease, suggesting the need for a “second hit” to trigger disease occurrence [21, 23].

In a murine model of malaria‐associated AKI, Possemiers et al. showed that glomerular collapse occurred in the absence of renal parasite sequestration and was instead driven by upregulation of interferon‐γ and TNF‐α [24]. Interferons can markedly upregulate APOL1 expression, and overexpression of APOL1 risk variants is particularly toxic to podocytes [23]. Acute *P. falciparum* infection may therefore act as a “second hit” in individuals carrying two APOL1 risk alleles, triggering interferon‐mediated podocyte injury and collapsing glomerulopathy [21].

This case shares many features, such as African ancestry, concomitant HIV infection, and severe AKI with need for KRT, with the cohort published by Amoura et al. APOL1 genotyping was not available in this case, which is a limitation of this report.

The prognosis of malaria‐associated collapsing glomerulopathy appears guarded. While complete kidney recovery is observed in up to 64% of cases of AKI or acute GN associated with *Plasmodium spp*. [25], the outcomes of collapsing glomerulopathy are notably worse. In the French cohort by Amoura et al., after a mean follow‐up of 23 months, 8 of 23 patients required long‐term KRT—including two who underwent kidney transplantation—and the mean eGFR among the remaining patients was only 51 mL/min/1.73 m^2^ [21]. Similarly, in this case, while the patient did not require long‐term KRT, there was only partial recovery of kidney function with the patient progressing to CKD G4A3.

The concurrent diagnosis of MM added further complexity to the differential diagnosis of this case. Cast nephropathy as the most common etiology for MM‐associated acute kidney injury was ruled out by the predominance of albuminuria [10] and the lack of light chain casts on biopsy. AL amyloidosis was a possible differential diagnosis but was eventually ruled out, as Congo red staining was negative on histological examination. Taken together, the clinical and histopathological findings were most consistent with malaria‐associated collapsing glomerulopathy.

This case illustrates the diagnostic challenge of AKI in critically ill patients with multiple comorbid conditions and highlights several practical implications. It underscores the importance of maintaining a broad differential in severe AKI, even when an apparent cause—such as sepsis—has been identified, as concurrent or alternative etiologies may be missed. Systematic screening for proteinuria in severe AKI can help detecting glomerular injury early, especially in patients of African ancestry who may carry APOL1 risk variants. Kidney biopsy remains invaluable in selected cases for establishing the correct diagnosis, guiding management, and informing prognosis.

**Nomenclature**
AKI: Acute kidney injury
APOL1: Apolipoprotein L1
ATN: Acute tubular necrosis
COVAN: SARS‐CoV‐2‐associated nephropathy
CRP: C‐reactive protein
eGFR: Estimated glomerular filtration rate
FSGS: Focal segmental glomerulosclerosis
HIV: Human immunodeficiency virus
HIVAN: HIV‐associated nephropathy
HUS: Hemolytic uremic syndrome
KDIGO: Kidney disease improving global outcomes
KRT: Kidney replacement therapy
MM: Multiple myeloma
PAS: Periodic acid–Schiff
UPCR: Urine protein‐to‐creatinine ratio
TMA: Thrombotic microangiopathy

## Author Contributions

Moritz Harenberg and Daniel Zickler analyzed and interpreted the patient data. Kerstin Amann performed the histological examination of the kidney and its interpretation in the clinical context. Moritz Harenberg drafted the manuscript with major contributions by Daniel Zickler and Kerstin Amann.

## Funding

The authors have nothing to report.

## Disclosure

All authors read and approved the final manuscript.

## Ethics Statement

The authors have nothing to report.

## Consent

All the patients allowed personal data processing and informed consent was obtained from all the individual participants included in this report.

## Conflicts of Interest

The authors declare no conflicts of interest.

## Data Availability

The data that support the findings of this study are available from the corresponding author upon reasonable request.
